# Plant miR6262 Modulates the Expression of Metabolic and Thermogenic Genes in Human Hepatocytes and Adipocytes

**DOI:** 10.3390/nu16183146

**Published:** 2024-09-18

**Authors:** Ester Díez-Sainz, Fermín I. Milagro, Paula Aranaz, José I. Riezu-Boj, Silvia Lorente-Cebrián

**Affiliations:** 1 Department of Nutrition, Food Science and Physiology, and Center for Nutrition Research, Faculty of Pharmacy and Nutrition, University of Navarra, 31008 Pamplona, Spain; ediezsainz@alumni.unav.es (E.D.-S.); paranaz@unav.es (P.A.); jiriezu@unav.es (J.I.R.-B.); 2 Navarra Institute for Health Research (IdiSNA), 31008 Pamplona, Spain; 3 Centro de Investigación Biomédica en Red Fisiopatología de la Obesidad y Nutrición (CIBERobn), Instituto de Salud Carlos III, 28029 Madrid, Spain; 4 Department of Pharmacology, Physiology and Legal and Forensic Medicine, Faculty of Health and Sport Science, University of Zaragoza, 50009 Zaragoza, Spain; slorentec@unizar.es; 5 Instituto Agroalimentario de Aragón-IA2, Universidad de Zaragoza- Centro de Investigación y Tecnología Agroalimentaria (CITA), 50013 Zaragoza, Spain; 6 Aragón Health Research Institute (IIS-Aragon), 50009 Zaragoza, Spain

**Keywords:** adipose tissue, browning, cross-kingdom regulation, diet, fatty liver, metabolism, NAFLD, plant microRNA, *RXRA*, steatosis

## Abstract

Background: Edible plants have been linked to the mitigation of metabolic disturbances in liver and adipose tissue, including the decrease of lipogenesis and the enhancement of lipolysis and adipocyte browning. In this context, plant microRNAs could be key bioactive molecules underlying the cross-kingdom beneficial effects of plants. This study sought to explore the impact of plant-derived microRNAs on the modulation of adipocyte and hepatocyte genes involved in metabolism and thermogenesis. Methods: Plant miR6262 was selected as a candidate from miRBase for the predicted effect on the regulation of human metabolic genes. Functional validation was conducted after transfection with plant miRNA mimics in HepG2 hepatocytes exposed to free fatty acids to mimic liver steatosis and hMADs cells differentiated into brown-like adipocytes. Results: miR6262 decreases the expression of the predicted target *RXRA* in the fatty acids-treated hepatocytes and in brown-like adipocytes and affects the expression profile of critical genes involved in metabolism and thermogenesis, including *PPARA*, *G6PC*, *SREBF1* (hepatocytes) and *CIDEA*, *CPT1M* and *PLIN1* (adipocytes). Nevertheless, plant miR6262 mimic transfections did not decrease hepatocyte lipid accumulation or stimulate adipocyte browning. Conclusions: these findings suggest that plant miR6262 could have a cross-kingdom regulation relevance through the modulation of human genes involved in lipid and glucose metabolism and thermogenesis in adipocytes and hepatocytes.

## 1. Introduction

Plant-based diets could contribute to energy homeostasis and exert beneficial properties towards the counteraction of white adipocyte tissue (WAT) and liver dysfunction, and brown adipose tissue (BAT) stimulation, emerging as a promising therapeutic approach to mitigate metabolic disturbances during metabolic disorders like non-alcoholic fatty liver disease (NAFLD), diabetes, and obesity [1,2,3,4]. A broad spectrum of foods from plants (including coffee, fruits, grains, herbal plants, legumes, olive oil, species, tea, and vegetables) have demonstrated therapeutic potential against liver homeostasis dysregulation, steatosis, and NAFLD development by decreasing lipid accumulation, inflammation, oxidative stress and apoptosis, and by modulating gut microbiota [2,5,6]. Additionally, plant-derived foods along with their bioactive components could alleviate adipose tissue dysfunction and promote browning [4,7,8]. These effects could be mediated through the inhibition of adipogenesis, lipid accumulation, cell proliferation, and pro-inflammatory adipokine release, as well as the promotion of mitochondrial biogenesis and activity, and thermogenesis [4,7,8].

The term plant-based diet encompasses any dietary pattern based on the consumption of plant foods while minimizing animal-based products [9]. Healthful plant-based diets consist of unprocessed plant products, such as vegetables, fruits, legumes, whole grains, nuts, and vegetable oils, minimizing or excluding processed foods and animal products [9]. Voluntary adherence to plant-based diets can be traced back to Egypt’s ancient civilizations, India, and Greece. Recognized figures such as Pythagoras, Gandhi, Albert Einstein, and Leonardo Da Vinci, popularized plant-based diets [10,11,12]. Throughout history, the promotion of ethical principles advocating non-violence towards animals fueled the switch from meat to plant-based products [10,11]. In recent years, interest in plant-based diets has continued to rise, driven not only by animal well-being concerns or spiritual beliefs but also by a broad range of factors, including human environmental footprint reduction and health improvement [11,12]. In this context, the expansion of scientific knowledge has played a key role in the widespread adoption of plant-based diets, as evidence has revealed an association between plant-based nutrition and reduced risk of several diseases such as diabetes, obesity, cardiovascular diseases, and cancer [10,12,13].The examination of plant-based diets effects on human health is an area of active research. Specifically, the discovery of plant-derived bioactive compounds and the elucidation of their mechanism of action is crucial to determining the therapeutic potential of plants in the alleviation of metabolic disturbances [14]. The benefits of plant consumption in the prevention and management of liver dysfunction and NAFLD have been largely linked to bioactive molecules such as terpenoids, polyphenols, polysaccharides, phytosterols, carotenoids, and omega-3 fatty acids [2,5,6]. The effects of plants on counteracting adipose tissue dysfunction and activating brown BAT could be mediated by bioactive compounds such as polyphenols, carotenoids, triterpenoids, and micronutrients [4,7,8,15]. Remarkably, it has been recently pointed out that microRNAs (miRNAs) may emerge as a new group of plant bioactive compounds with pivotal biological roles in animals [16,17].

Edible plant-based foods contain a wide variety of miRNAs that can withstand cooking procedures [18,19]. MiRNAs are short RNA species that do not encode proteins but are essential for the control of gene expression post-transcriptionally [20,21]. MiRNAs are pivotal within the plant kingdom, participating in a plethora of vital functions, including metabolism [22], stress responses [23], and growth [24]. A major area of interest is the significant involvement of plant miRNAs. Of particular interest is the role of plant miRNAs in cross-kingdom communication, since they can interact with mammalian genes to modulate processes such as cell death and proliferation [25,26,27], immune system responses, viral infections [28,29,30,31,32,33], and metabolism [34,35,36,37,38,39]. Although the bioavailability of plant miRNAs in animals is still a topic of debate due to contradictory findings [18,40,41], extensive evidence has highlighted their therapeutic potential against several diseases, including cancer, pulmonary fibrosis, viral infections such as COVID-19 and influenza, and metabolic disorders [29,30,36,38,42,43]. Therefore, miRNAs from diet might be an underlying factor in diet-induced epigenetic changes that influence the expression of genes involved in maintaining host homeostasis.

Despite the recognized therapeutic potential of plant-based diets in preventing and/or alleviating metabolic disturbances (such as restoring hepatocyte and adipocyte metabolism, inhibiting liver steatosis, and activating BAT [4,6,44]), it remains unclear whether plant miRNAs could contribute to these beneficial effects by interacting with metabolic genes in human hepatocytes and adipocytes. Unravelling the specific mechanism underlying the cross-kingdom activities of miRNAs derived from plants would be key to elucidating their potential therapeutic efficacy against human diseases. Thus, the focus of this study was to investigate the impact of plant miRNAs on the modulation of metabolic and thermogenic-related genes in an in vitro model of liver steatosis and in brown-like adipocytes.

## 2. Materials and Methods

### 2.1. In Silico Identification of Potential Interactions between Plant miRNAs and Human Genes

Bioinformatic identification of candidate human target genes of plant miRNAs was carried out as previously detailed [39]. Briefly, *Prunus persica* miRNA sequences from miRBase (https://www.mirbase.org/; accessed on 12 April 2022) were selected to predict human targets using psRNATarget (https://www.zhaolab.org/psRNATarget/; accessed on 13 April 2022) [45] and TAPIR (http://bioinformatics.psb.ugent.be/webtools/tapir/; accessed on 13 April 2022), with default parameters applied [46]. Gene ontology (GO) enrichment analyses of predicted human targets were performed using GeneCodis (https://GeneCodis.genyo.es/; accessed on 15 April 2022) [47]. Additionally, a bibliographic review was performed in PubMed database (https://pubmed.ncbi.nlm.nih.gov/; accessed on 15–28 April 2022), focusing exclusively on the predicted target genes that were consistently identified across the three abovementioned prediction algorithms. For subsequent in vitro studies, only miRNAs with predicted targets linked to liver/adipose tissue metabolism, adipocyte browning, and/or NAFLD were chosen.

### 2.2. Cell Culture and Transfections with Plan miRNA Mimics

The establishment of the human multipotent adipose-derived stem cells hMADS has been previously described [48,49]. hMADS cells were cultured in a 37 °C incubator with 5% CO_2,_ using DMEM low glucose (1 g/L) L-GlutaMAX (Gibco. Thermo Fisher Scientific Inc., Waltham, MA, USA), 10% fetal bovine serum (FBS; Eurobio, Les Ulis, France), 1% penicillin-streptomycin solution (P/S; Gibco. Thermo Fisher Scientific Inc.) and 15 mM HEPES (4-(2-hydroxyethyl)-1-piperazineethanesulfonic acid; Gibco. Thermo Fisher Scientific Inc.). In addition, human fibroblast growth factor 2 (hFGF2; Peprotech. Thermo Fisher Scientific Inc.), which is a key mitogenic factor to promote hMADS proliferation [50], was freshly added at 2.5 ng/mL to the medium before each use.

For the experiments, undifferentiated hMADS cells were plated at a density of 25,000 cells per well in 12 well-plates for gene expression assays and in 6 well-plates (50,000 cells/well) for western blot and mitochondrial DNA quantification assays, in DMEM low glucose (1 g/L) L-GlutaMAX supplemented with 10% FBS, 1% P/S, 15 mM HEPES and 2.5 ng/mL hFGF2 freshly added prior each use. Once cells reached confluence (defined as day −2 of differentiation), hFGF2 was removed to arrest cell cycle. After 2-day post-confluence, cells underwent differentiation into adipocytes for 18 days and were maintained using DMEM low glucose (1 g/L) L-GlutaMAX mixed 1:1 with Ham’s F12 with L-glutamine (Gibco. Thermo Fisher Scientific Inc.) and supplemented with 15 mM HEPES, 1% P/S, 10 nM insulin (Invitrogen. Thermo Fisher Scientific Inc.), 10 µg/mL transferrin (Tf; Sigma-Aldrich, San Luis, MO, USA) and 0.2 nM triiodothyronine (T3; Sigma-Aldrich). From day 0 to day 4 of differentiation, cells were also exposed to 1 µM dexamethasone (Dex; Sigma-Aldrich) and 500 µM isobutyl-methylxanthine (IBMX; Sigma-Aldrich). The PPARγ agonist rosiglitazone (Rosi; Cayman Chemical Company, Ann Arbor, MI, USA) was incorporated at 100 nM from day 2 to 9 to promote white adipocyte differentiation [51,52]. Additionally, the browning process was induced by treating cells with 100 nM Rosi between days 14 and 18, thus stimulating the conversion of white adipocytes into thermogenic brite adipocytes [51,52]. Media was changed every other day and insulin, Tf, T3, Dex, IBMX and Rosi were added immediately before each use.

Transfections of hMADS cells were performed between day 10–12 of differentiation using Lipofectamine RNAiMAX Reagent and 25 nM of mirVana™ miRNA mimics (Thermo Fisher Scientific Inc.), including a scramble sequence as negative control (mirVana™ miRNA Mimic, Negative Control #1) and miR6262 (5′-UCUUUAGAAAGUUAGAAUUGU-3′; assay ID MC29621). Cell medium was changed to DMEM low glucose (1g/l) L-GlutaMAX/Ham’s F12 with L-glutamine supplemented with 15 mM HEPES, 1% P/S, 10 nM insulin, 10 µg/mL Tf and 0.2 nM T3 (900 µL/well in 6 well/plates, 450 µL/well in 12 well/plates). In Opti-MEM I Reduced Serum Medium (Gibco. Thermo Fisher Scientific Inc.), lipofectamine and miRNA mimics were diluted in a 1:1 ratio and incubated at room temperature (RT) for 10 min. The resulting lipofectamine-miRNA mimic complexes were then added to the wells at 300 µL per well for 6-well plates and 150 µL per well of 12-well plates. Of note, lipofectamine volumes were the following: 9 µL per well for 6-well plates (in a volume of 1200 µL) and 4.5 µL per well for 12-well plates (in a volume of 600 µL). Cells medium was changed after 24 h of transfections and cells continued the differentiation process until day 18 for gene expression, Western Blot, and mitochondrial DNA quantification assays.

Cell culture, miRNA mimic transfection and lipid accumulation induction in the human hepatoma HepG2 cell line (American Type Culture Collection, ATCC^®^ HB-8065™; Manassas, VA, USA) were conducted as previously detailed [39]. HepG2 cells were transfected using the reverse method with 50 nM of the same mirVana™ miRNA mimics (Thermo Fisher Scientific Inc.) used for hMADS transfections.

### 2.3. RNA Purification and Gene Expression Assays

mRNA expression was evaluated in hMADS differentiated into white and brite adipocytes that were transfected at day 10–12 of differentiation and collected at day 18. Cells were detached mechanically with a scraper in TRI-Reagent (Molecular Research Center Inc., Cincinnati, OH, USA) and RNA isolation was performed according to the manufacturer’s instructions. The purity (260/280 ratio) and concentration of RNA were assessed using a NanoDrop 2000 (Thermo Fisher Scientific Inc.). In addition, quality control of RNA was tested by agarose gel electrophoresis analysis: RNA was stained with GelRed Nucleic Acid Gel Stain (Biotium Inc., Fremont, CA, USA) diluted in BlueJuice Gel Loading Buffer (Invitrogen. Thermo Fisher Scientific Inc.), electrophoresis was performed in 1% agarose gel immersed in 0.5 X Tris-acetate-EDTA (TAE) using a Mupid-exU system (Takara; Tokyo, Japan) at 100 V for 30 min, and visualised in a Gene Flash UV imager (Syngene Bio imaging, Cambridge, UK).

Reverse-transcription of hMADS RNA was conducted according to the manufacturer’s guidelines as follows: RNA (1 µg) was mixed with RQ1 RNase-Free DNAse (Promega, Madison, WI, USA), RNAsin (Promega) and Reaction Buffer from M-MLV-RT kit (Promega), was incubated for 15 min at 37 °C, 5 min at 75 °C, and 3 min at 4 °C. Samples were incubated with Primer “random” (Roche, Basel, Switzerland) for 5 min at 70 °C. dNTP Mix (Set of dATP, dCTP, dGTP, dTTP; Promega), M-MLV-RT and Reaction Buffer from M-MLV-RT kit (Promega) were added to the samples and incubated for 60 min at 37 °C. The reactions were carried out in a Bioer GeneExplorer Thermal Cycler (Bioer Technology, Hangzhou, China). Quantitative PCR (qPCR) was performed with hMADS cDNA diluted 1/5, ONEGreen FAST qPCR Premix (Ozyme, Saint-Cyr-l’École, France) and Integrated DNA Technologies predesigned assays (IDT; Coralville, IA, USA) (Table 1), and oligonucleotide sequences generated with Primer Express Software (Perkin Elmer and Analytical Sciences, Boston, MA, USA; http://www.perkinelmer.com) (Table 2). Reactions were performed on a StepOnePlus Real-Time PCR System (Applied Biosystems. Thermo Fisher Scientific Inc.), applying the following cycling conditions: 95 °C for 30 s; 40 cycles at 95 °C for 5 s and 60 °C for 34 s; a melt curve stage of 95 °C for 15 s, 60 °C 1 min, 95 °C 15 s. Normalization of gene expression levels (∆Cq) was performed using as housekeeping control the gene expression of *36B4* (also termed as Ribosomal Protein Lateral Stalk Subunit P0, *RPLP0*) [53]. Relative gene expression was determined by stabilizing comparisons with the scramble-sequence-transfected cells differentiated to brite adipocytes (brite negative control) using the 2^−∆∆Cq^ method [54,55].

The procedures for RNA purification and gene expression evaluation in HepG2 cells were carried out according to previously described methods [39]. Additionally, transfection efficiency was evaluated in HepG2 cells using the miRCURY LNA miRNA PCR Assay (Qiagen, Hilden, Germany) miR6262 (5′-UCUUUAGAAAGUUAGAAUUGU-3′; GeneGlobe ID: YP02112521) as previously detailed [39].

### 2.4. Cytotoxicity Assays and Intracellular Triglyceride Quantification in HepG2 Cells

Cell viability and intracellular triglyceride quantification were evaluated in HepG2 transfected for two days, both untreated and treated with free fatty acids (FFA) for 3 h as previously described [39].

### 2.5. Western Blot and Mitochondrial DNA Quantification Analysis in hMADS Cells

Protein expression of the key marker of the browning process UCP-1 [52], was evaluated in hMDAS cells differentiated into white and brite adipocytes that were transfected at 10–12 of differentiation and collected at day 18. Protein extraction and quantification were conducted as indicated: cells were first washed with PBS at 4 °C, detached mechanically with a scraper in protein lysis buffer, and sonicated 3 times (5–10 sec each) at 4 °C using a Sonifier ultrasonic homogenizer (Branson, Brookfield, CT, USA). Lysis buffer consisted of 25 mM Tris-Cl (pH 7.4), 100 mM NaCl, 1 mM EDTA, 0.5% Triton X-100, 0.5% Nonidet P40, 1X protease inhibitor cocktail (Roche Life Sciences, Indianapolis, IN, USA). Samples were subjected to centrifugation at 14,000× *g* for 10 min at 4 °C and supernatant was collected. Protein concentration was measured using the Pierce BCA Protein Assay kit (Thermo Fisher Scientific Inc.), with absorbance recorded at 560 nm in an iMark™ Microplate Absorbance Reader (Bio-Rad, Hercules, CA, USA). Immediately before electrophoresis, protein samples were diluted at a final concentration of 40 µg per sample in lysis buffer with 1% loading buffer Laemmli (250 mM Tris-HCl pH 6.8, 8% Sodium dodecyl-sulphate, 10% glycerol, 0.01% bromophenol blue) and 0.1 M DTT (dithiothreitol; Thermo Fisher Scientific Inc.) and heated at 95 °C for 5 min. Sodium dodecyl-sulphate polyacrylamide gel electrophoresis (SDS-PAGE) was conducted with 40 µg of protein per sample using gradients gels (11%), in a power supply Hoefer EPS 2A200 (Hoefer Inc., Holliston, MA, USA) at 70 V for 30 min and 120 V for 2 h. Proteins were transferred from gels into polyvinylidene fluoride (PVDF) membranes using a liquid transfer system Mini Trans-Blot^®^ Cell (Bio-Rad) at 110 V, 200 mA for 1.5 h at 4 °C. Membranes were blocked with 5% skimmed milk in 1X tris Buffered Saline with 0.05% tween (TBST) at RT for 1 h and washed with TBST. Membranes were subsequently incubated at 4 °C overnight (ON) with primary antibodies from rabbit: anti-UCP1 (#Ab10983, Abcam, Waltham, MA, USA) and anti-TBP (anti-TATA-box binding protein; #44059, Cell Signaling Technology, Danvers, MA, USA) diluted 1:1000. Membranes were washed with TBST and Horseradish peroxidase (HRP)-conjugated anti-rabbit antibody (Promega) diluted 1:10000 was incubated at RT for 1 h to detect primary antibodies. After washing membranes with TBST, HRP-signal detection was revealed with Clarity Western enhanced chemiluminescence (ECL) Substrate (Bio-Rad) in a luminescent imager analyzer (Amersham Imager 600; GE Healthcare, Chicago, IL, USA). Signal intensity of the protein bands was quantified with ImageJ software (Version 1.52a). UCP-1 signal intensity was normalized to that of TBP protein and results were expressed as the % relative protein concentration by establishing comparison with brite negative control.

White and brite adipocyte hMDAS cells (transfected at 10–12 of differentiation and collected at day 18) were used to evaluate the impact of plant miR6262 on mitochondriogenesis, which is associated with the browning process [56,57]. Since mitochondria biogenesis is characterized by DNA mitochondrial content increase, the levels of the mitochondrial gene NADH dehydrogenase Subunit 1 (*NADHdS1*) were quantified by qPCR and normalized with those of the nuclear gene Lipoprotein Lipase (*LPL*) [57,58]. DNA was isolated with PureLink Genomic DNA Mini Kit (Invitrogen. Thermo Fisher Scientific Inc.) following the manufacturer’s protocol, and quantity and quality of DNA was tested in a NanoDrop 2000 (Thermo Fisher Scientific Inc.). qPCR was performed with 2 ng DNA, ONEGreen FAST qPCR Premix (Ozyme) and assays designed with Primer Express Software (Perkin Elmer and Analytical Sciences) to measure *NADHdS1* and *LPL* gene expression levels (Table 3). qPCR reactions were conducted in a StepOnePlus Real-Time PCR System (Applied Biosystems. Thermo Fisher Scientific Inc.): 95 °C 5 min; 40 cycles at 95 °C 10 s and 60 °C 20 s; a melt curve stage of 95 °C 15 s, 60 °C 1 min, 95 °C 15 s. *NADHdS1* expression levels were normalized to that of *LPL* and relative gene expression was determined with the 2^−∆∆Cq^ method by stabilizing comparison with brite negative control [54,55].

### 2.6. Statistical Analysis

GraphPad Prism 6.0 for Windows (GraphPad Software Inc., La Jolla, CA, USA) was used for statistical analysis. The two-tailed Student’s t-test was applied to compare the experimental group (plant miR6262 transfected cells) with the negative control (scramble- sequence-transfected cells). Statistical significance was determined at a *p*-value < 0.05.

## 3. Results

### 3.1. Selection of Plant miRNAs with Putative Human Target Genes Related to Metabolism

The selection of plant miRNAs that could potentially target metabolic human genes were conducted applying the following criteria: (1) A reference genome was chosen for the in-silico analyses, which is a common strategy for plant miRNA identification due to their widespread conservation across different plant species [59,60]. In particular, *Prunus persica* was chosen for plant miRNA selection because of the following points: its high-quality genome assembly and characterization; its comprehensively well-annotated miRNA profile; and its status as a globally consumed edible fruit (https://mirbase.org/results/?query=prunus+persica) (https://plants.ensembl.org/Prunus_persica/Info/Index) (accessed on 12 April 2022) [61]. Thus, a dataset of 214 different miRNA sequences from *Prunus persica* were retrieved from miRBase. (2) Prediction of potential human targets for plant miRNAs was conducted using the prediction tools TAPIR and psRNATarget. The criteria for selecting miRNA-mRNA pairs for further analysis required choosing only miRNA sequences that showed consistent target prediction across both TAPIR and psRNATarget (V1 and V2 scoring schemas) to enhance prediction accuracy [62]. This selection resulted in 163 miRNA-mRNA pairs, which included 91 distinct miRNAs, and 112 different transcripts derived from 100 distinct genes. (3) Lastly, the criteria for selecting candidate miRNA-mRNA pairs for in vitro studies involved identifying genes from the set of 100 that were associated with liver/adipocyte metabolism, NAFLD and/or adipocyte browning, conducing GO analysis and a literature review. As a result of the bioinformatic analysis, miR6262 (from *Prunus persica*), which had RXRA (Retinoid X Receptor Alpha) as a potential target gene, was selected for subsequent in vitro studies since it displayed all the selection criteria: (1) its putative targets were predicted by psRNATarget (scoring schemas V1 and V2) and TAPIR, and (2) its potential target was involved in metabolism.

Upon detailed examination of the bioinformatic results for miR6262, miRNA-human target gene predictions revealed 16 (psRNATarget scoring schema V1), 24 (psRNATarget scoring schema V2), and 7 (TAPIR) predicted targets of plant miR6262 (Table 4). Overall, 25 different transcripts (24 different genes) were identified as putative targets of plant miR6262. Then, GO analysis to search enriched pathways was conducted with the 24 predicted target genes (*OSBPL8*, *PAN3*, *ATP6V1C1*, *RXRA*, *GPR137B*, *PRDM15*, *EPT1*, *CLEC12B*, *NCOA7*, *ATG12*, *PPARGC1B*, *GPR4*, *CNEP1R1*, *PALM2-AKAP2*, *ZNF8*, *ST8SIA1*, *BBS10*, *RAP2C*, *GOLT1B*, *IL6R*, *WNT5A*, *ABCB10*, *GLRA3*, *AK4*), all of which were reported as annotated inputs by GeneCodis4. A wide range of enrichment pathways were identified, including regulation of vascular permeability (*GPR4*), hepatic immune response (*IL6R*), and negative regulation of cellular response to oxidative stress (*NCOA7*) (Table S1). Nevertheless, only six transcripts were common targets to all prediction algorithms used, which correspond to the genes *OSBPL8* (Oxysterol Binding Protein Like 8; transcript NM_020841), *ATP6V1C1* (ATPase H+ Transporting V1 Subunit C1; transcript NM_001695), *RXRA* (transcript NM_002957), *GPR137B* (G Protein-Coupled Receptor 137B; transcript NM_003272), *CLEC12B* (C-Type Lectin Domain Family 12 Member B; transcript NM_205852), and *PALM2-AKAP2* (PALM2 And AKAP2 Fusion; transcript NM_007203) (Table 4, Figure S1). Thus, to choose the most robust miRNA-mRNA interaction predictions, these were the only putative target genes of miR6262 used for bibliographic research to identify functions related to metabolism. Notably, RXR receptors play a crucial role in hepatic lipid metabolism regulation, adipogenesis, and adipocyte lipogenesis, and their inhibition has been linked to a reduced risk of diet-induced obesity and related comorbidities such as diabetes [63,64,65,66]. Thus, miR6262 and its predicted target *RXRA* were selected as candidates for in vitro validation. We addressed the potential inhibitory effect of miR6262 on *RXRA* gene expression and evaluated the impact of miR6262 on key metabolic genes in hepatocytes (HepG2) untreated and exposed to free fatty acids (FFA) to mimic hepatic steatosis, and in brite adipocytes (hMADS cells).

### 3.2. Plant miR6262 Was Detected in Transfected Hepatocytes and Did Not Induce Cytotoxicity Effects

The efficiency of the transfection procedure was determined 6 h after transfection of HepG2 cells with 50 nM of plant miR6262 mimic and the negative control (Figure S2). Positive expression of plant miR6262 mimic were reported (Cq values between 20–23), confirming the efficiency of the transfections. Negative expression of plant miR6262 was reported in control cells, suggesting the absence of a homologous human sequence of plant miR6262 in human hepatocytes.

Cytotoxicity assays were conducted 48 h after transfection of HepG2 with plant miR6262 mimic and the negative control, untreated and treated with FFA (3 h) (Figure S3). Transfection with plant miR6262 did not induce cytotoxic effects in untreated (Figure S3a) and FFA-treated (Figure S3b) hepatocytes since no decrease in cellular viability was reported.

### 3.3. Plant miR6262 Regulated the Expression of the Predicted Target RXRA and Metabolic-Related Genes in Hepatocytes

The impact of plant miR6262 on the expression of the predicted target RXRA, as well as transcription factors (*PPARA*, *FOXO1*, *SREBF1*) and functional protein-coding genes (*MAPKAPK2*, *QKI*, *FASN*, *ACOX1*, *G6PC*, *GSK3B*) involved in lipid and glucose metabolism, was evaluated after 48 h of transfection in HepG2 untreated and treated with FFA for 3 h. The effect of plant miR6262 on the mRNA expression of the putative target (RXRA), along with crucial genes involved in glucose and lipid metabolism (the transcription factors *FOXO1*, *PPARA*, and *SREBF1*; and the functional protein-coding genes *ACOX1*, *FASN*, *G6PC*, *GSK3B*, *QKI*, *MAPKAPK2*) was assessed 48 h post-transfection in hepatocytes (HepG2 cells) both untreated and exposed to FFA for 3 h.

**Figure 1:**
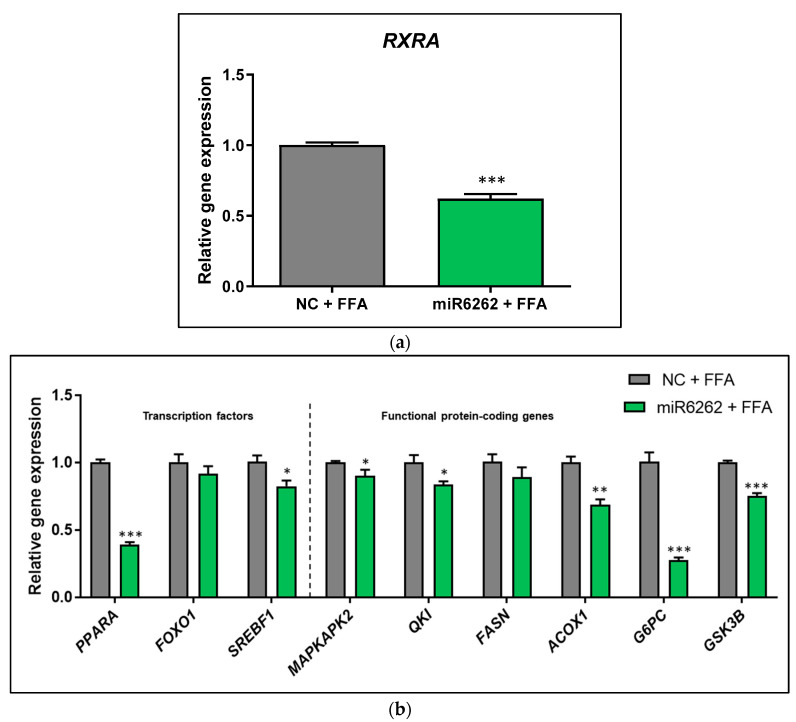
Evaluation of gene expression in hepatocytes after transfection with plant miR6262 mimic and treatment with free fatty acids to mimic in vitro liver steatosis. (**a**) mRNA expression of the predicted target *RXRA*. (**b**) mRNA expression of transcription factors (*PPARA*, *FOXO1*, and *SREBF1*) and functional protein-coding genes (*MAPKAPK2*, *QKI*, *FASN*, *ACOX1*, *G6PC*, and *GSK3B*) associated with lipid and glucose metabolism. Reverse-transfection of HepG2 cells was conducted with the plant miR6262 mirVana mimic (5′-UCUUUAGAAAGUUAGAAUUGU-3′) and a negative control (a scramble sequence) at 50 nM. At 48 h post-transfection, cells were exposed to free fatty acids (a mixture of oleic and palmitic acids at 0.5 M and a 2:1 ratio) for 3 h. Quantification of mRNA expression levels was evaluated by qPCR. *TBP* was selected as housekeeping gene to normalize Cq values, which were expressed relative to the free FFA-treated negative control, applying the 2*^−^*^ΔΔCt^ method. Results are shown as relative gene expression mean relative gene expression mean ± standard error of the mean (SEM) (n = 4–5). *p*-value: * *p* < 0.05, ** *p* < 0.01, *** *p* < 0.001. Abbreviations: NC (negative control), FFA (free fatty acids).

In the in vitro model of liver steatosis (HepG2 cells exposed to FFA), the plant miRNA-human target gene bioinformatic prediction was validated, reporting a marked downregulation of the putative target *RXRA* (−38.05% ± 3.91; *p* < 0.001) as compared to control cells (Figure 1a). FFA-exposed HepG2 also showed a reduction in the mRNA levels of *PPARA* (−61.25% ± 2.86; *p* < 0.001), *SREBF1* (−18.10% ± 6.49; *p* < 0.05), *MAPKAPK2* (−10.60% ± 3.99; *p* < 0.05), *QKI* (−16.65% ± 5.38; *p* < 0.05), *ACOX1* (−31.65% ± 5.92; *p* < 0.01), *G6PC* (−73.35% ± 6.49; *p* < 0.001), and *GSK3B* (−24.80% ± 2.65; *p* < 0.001), whereas any significant differences were found in FOXO1 mRNA expression (Figure 1b).

Basal (non-FFA-treated HepG2) transfected with plant miR6262 mimic also exhibited a decrease in the mRNA levels of the predicted target *RXRA* (−31.14% ± 3.62; *p* < 0.001), (Figure S4a), which was accompanied by an mRNA downregulation of *PPARA* (−39.67% ± 7.54; *p* < 0.05), *SREBF1* (−26.86% ± 5.82; *p* < 0.001), *FASN* (−21.57% ± 7.86; *p* < 0.05), and *G6PC* (−66.86% ± 5.23; *p* < 0.001), and an increase of *FOXO1* (29.71% ± 11.97; *p* < 0.05), mRNA levels relative to the negative control (Figure S4b). No significant changes were detected in *MAPKAK2*, *QKI*, *ACOX1*, and *GSK3B* mRNA of untreated HepG2 cells (Figure S4b).

### 3.4. Plant miR6262 Did Not Attenuate Lipid Accumulation in an In Vitro Hepatocyte Human Cell Model of Liver Steatosis

**Figure 2:**
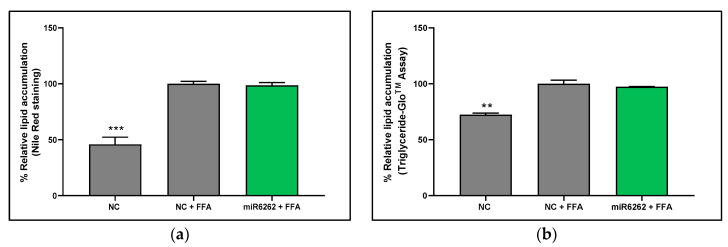
Quantification of intracellular lipid levels in hepatocytes after transfection with plant miR6262 mimic. (**a**) Intracellular triglycerides levels measured by Nile Red staining. (**b**) Intracellular triglycerides levels measured by Triglyceride-Glo^TM^ Assay. Reverse-transfection of HepG2 cells was conducted with the plant miR6262 mirVana mimic (5′-UCUUUAGAAAGUUAGAAUUGU-3′) and a negative control (a scramble sequence) at 50 nM. At 48 h post-transfection, cells were exposed to free fatty acids (a mixture of oleic and palmitic acids at 0.5 M and a 2:1 ratio) for 3 h. Scramble-sequence-transfected cells, untreated with FFA, served as positive control. Results are expressed as the percentage of lipid accumulation, reflecting the mean ± standard error of the mean (SEM) of the lipid content versus to FFA-treated negative control (Nile red staining: n = 6–7; Triglyceride-Glo^TM^ assay: n = 3). Statistical significance was assessed by comparing miR6262 transfected cells and untreated negative control to the FFA-treated negative control. *p*-value ** *p* < 0.01, *** *p* < 0.001. Abbreviations: NC (negative control), FFA (free fatty acids).

Lipid content within HepG2 cells was quantified 48 h post-miRNA mimic transfection with miRNA mimics, comparing untreated cells with those exposed to FFA for 3 h. The in vitro model of liver steatosis was validated by two independent methods: an increase of the lipid content in HepG2 cells exposed to FFA relative to untreated cells was detected with both Nile Red staining (54.15% ± 6.28; *p* < 0.001) (Figure 2a) and Triglyceride-Glo^TM^ Assay (27.63% ± 3.48; *p* < 0.01) (Figure 2b). However, the plant miR6262 mimic did not affect lipid accumulation in FFA-treated hepatocytes when compared to negative control exposed to FFA (Figure 2b).

### 3.5. Plant miR6262 Regulated the Expression of the Predicted Target RXRA and Metabolic-Related Genes in hMADS Cells Differentiated into Brite Adipocytes

The impact of plant miR6262 on the miRNA expression levels of the predicted target gene as well as genes associated with metabolism, adipogenesis, and adipocyte thermogenesis and browning, was evaluated in hMADS differentiated into brite adipocytes. In particular, we analyzed the mRNA expression levels of the predicted target gene *RXRA*, along with the following genes: *UCP1*, *FABP4*, *CIDEA*, *PPARG*, *HSL*, *ATGL*, *PLIN1*, *ACOX1*, *ADRB3*, *COL1A1*, *MAPKAPK2*, and *CPT1M*.

**Figure 3:**
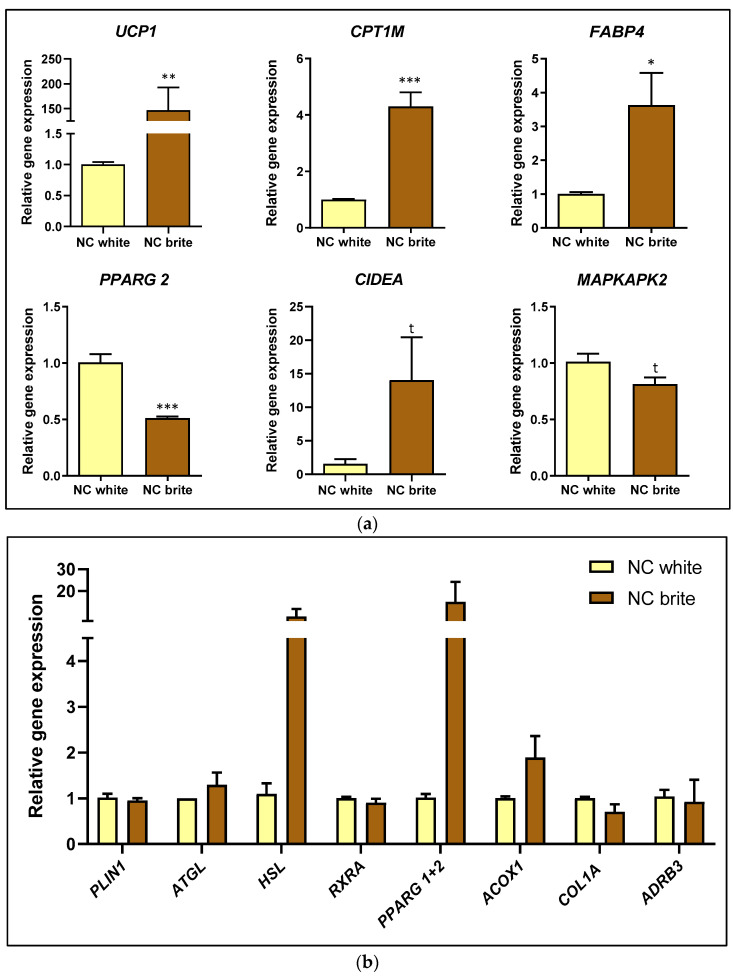
Evaluation of gene expression in hMADS cells differentiated into white and brite adipocytes. (**a**) mRNA levels of *UCP1*, *CPT1M*, *FABP4*, *PARG2*, *CIDEA*, and *MAPKAPK2*. (**b**) mRNA levels of *PLIN1*, *ATGL*, *HSL*, *RXRA*, *PPARG 1+2*, *ACOX1*, *COL1A*, and *ADRB3*. Transfection of hMADS cells was conducted with 25 nM of the negative control (a scramble sequence) at day 10–12 of differentiation. Stimulation of the conversion of white adipocytes into brite adipocytes (browning process) was conducted from day 14 to day 18 of differentiation. Quantification of mRNA expression levels was conducted by qPCR in white and brite adipocytes. 36B4 was selected as housekeeping gene to normalize Cq values, which were expressed relative to the white negative control, applying the 2^-ΔΔCt^ method. Results are depicted as the relative gene expression mean ± standard error of the mean (SEM) (n = 4–6). *p*-value: * *p* < 0.05, ** *p* < 0.01, *** *p* < 0.001, t (CIDEA) = 0.0813; t (MAPKAPK2) = 0.0551. Abbreviations: NC (negative control).

Firstly, the efficiency of adipocyte browning stimulation was evaluated in adipocytes transfected with a scramble sequence by establishing comparisons with scramble-sequence-transfected white adipocytes (Figure 3). In brite control cells, it was detected a marked up-regulation of the thermogenic markers *UCP1* (fold change 145.8 ± 45.73; *p* < 0.01) and *CPT1M* (fold change 3.30 ± 0.50; *p* < 0.001) (Figure 3a). Furthermore, the mRNA levels of the mature adipocyte marker FABP4 increased (fold change 2.62 ± 0.95; *p* < 0.05) in brite adipocytes relative to white adipocytes (Figure 3a). Stimulation of the browning process decreased the mRNA expression of *PPARG2* (fold change −0.50 ± 0.07; *p* < 0.001), and a tendency was reported towards the enhancement of *CIDEA* (fold change 12.50 ± 6.45; t = 0.0813) and the decrease of *MAPKAPK2* (fold change −0.20 ± 0.09; t = 0.0551) mRNA expression (Figure 3a). No changes at the mRNA levels of *PLIN1*, *ATGL*, *HSL*, *RXRA*, *PPARG* 1+2; *ACOX1*, *COL1A* and *ADRB3* were reported in brite adipocytes (Figure 3b).

**Figure 4:**
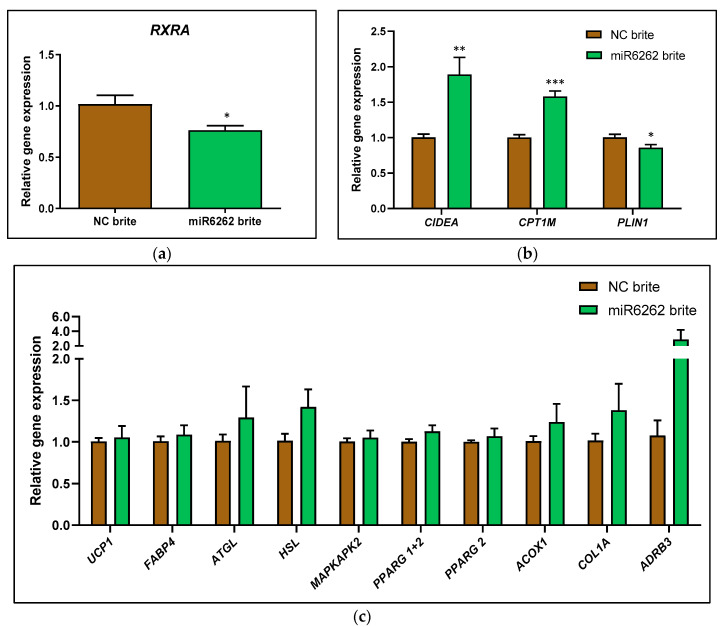
Evaluation of gene expression in hMADS cells after transfection with plant miR6262 mimic and differentiation into brite adipocytes. (**a**) mRNA expression of the predicted target *RXRA*. (**b**) mRNA expression of *CIDEA*, *CPT1M*, and *PLIN1*. (**c**) mRNA levels of *UCP1*, *FABP4*, *ATGL*, *HSL*, *MAPKAPK2*, *PPARG 1+2*, *PPARG2*, *ACOX1*, *COL1A*, and *ADRB3*. Transfection of hMADS cells was performed with 25 nM of the plant miR6262 mirVana mimic (5′-UCUUUAGAAAGUUAGAAUUGU-3′) and a negative control (a scramble sequence) at day 10–12 of differentiation. Stimulation of the conversion of white adipocytes into brite adipocytes (browning process) was conducted between day 14 to day 18 of differentiation. Quantification of mRNA expression levels was evaluated by qPCR in white and brite adipocytes. 36B4 was selected as housekeeping gene to normalize Cq values, which were expressed relative to the brite negative control, applying the 2^−ΔΔCt^ method. Results are shown as the relative gene expression mean ± standard error of the mean (SEM) (n = 4–6). *p*-value: * *p* < 0.05, ** *p* < 0.01, *** *p* < 0.001. Abbreviations: NC (negative control).

On the other hand, plant miR6262 mimic transfection decreased the mRNA expression of the predicted target *RXRA* (−25.50% ± 9.74; *p* < 0.05) in brite adipocytes as compared to brite control cells (Figure 4a), which was accompanied by an increase of CIDEA (88.50% ± 24.85; *p* < 0.01) and *CPT1M* (57.67% ± 8.86; *p* < 0.001), and a decrease of PLIN1 (−14.50% ± 6.04; *p* < 0.05) mRNA levels (Figure 4b). Plant miR6262 mimic did not modify the mRNA levels of *UCP1*, *FABP4*, *ATLG*, *HSL*, *MAPKAPK2*, *PPARG 1+2*, *PPARG 2*, *ACOX1*, *COL1A*, and *ADRB3* when compared to brite control cells (Figure 4c).

### 3.6. Plant miR6262 Did Not Have an Impact on UCP-1 Protein Expression and the Mitochondrial DNA Content in hMADS Cells Differentiated into Brite Adipocytes

UCP-1 protein expression levels and quantification of mitochondrial DNA, which correlated with mitochondriogenesis, were selected as markers of adipocyte browning to investigate the functional impact of plant miR6262 on this process.

**Figure 5:**
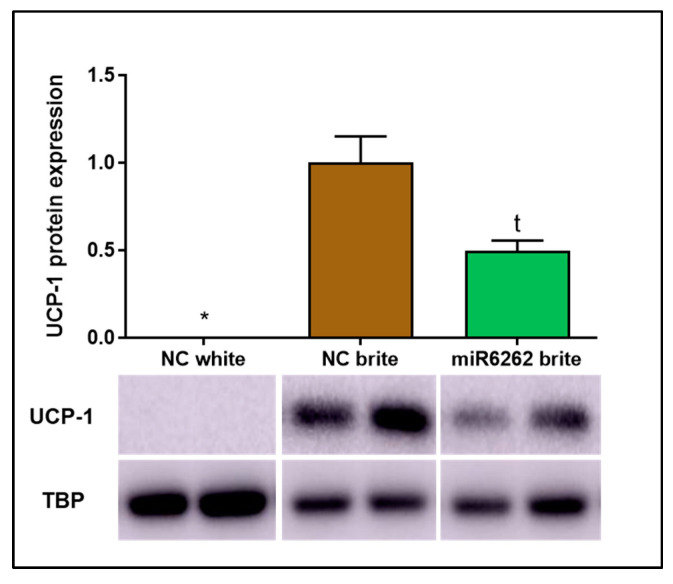
UCP-1 protein expression in hMADS cells differentiated into brite and white adipocytes and plant miR6262 mimic transfected brite adipocytes. Transfection of hMADS cells were conducted with 25 nM of the mirVana mimic plant miR6262 (5′-UCUUUAGAAAGUUAGAAUUGU-3′) and a negative control (scramble sequence) at day 10–12 of differentiation. Stimulation of the conversion of white adipocytes in brite adipocytes (browning process) was conducted between days 14 and 18 of differentiation. UCP-1 protein levels were quantified by western blot and the signal intensity of the protein bands was measured with ImageJ software (version 1.52a). UCP-1 signal intensity was normalized to that of TBP and results are presented as UCP-1 protein expression levels relative to brite negative control ± standard error of the mean (SEM) (n = 2). *p*-value: * *p* < 0.05; t = 0.0883. Abbreviations: NC (negative control).

Consistent with gene expression data and supporting the efficacy of the browning process induction, UCP-1 protein levels were increased in brite adipocytes when compared to white adipocytes, where UCP-1 protein expression was undetectable (Figure 5). A tendency towards the decrease of UCP-1 expression was reported in brite adipocytes transfected with plant miR6262 mimic in comparison to brite adipocyte control (−50.33% ± 16.03; t = 0.0883) (Figure 5).

**Figure 6:**
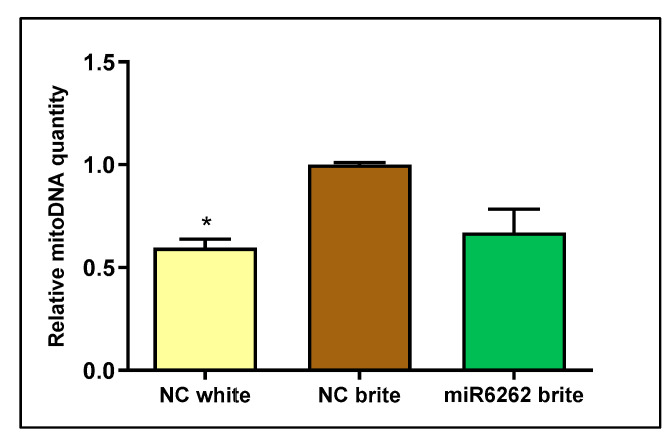
Mitochondrial DNA quantification in hMADS cells differentiated into brite and white adipocytes and plant miR6262 mimic transfected brite adipocytes. Transfection of hMADS cells was performed with 25 nM of the plant miR6262 mirVana mimic (5′-UCUUUAGAAAGUUAGAAUUGU-3′) and a negative control (a scramble sequence) at day 10–12 of differentiation. Stimulation of the conversion of white adipocytes in brite adipocytes (browning process) was conducted between days 14 and 18 of differentiation. DNA expression levels were analyzed in white and brite adipocytes by qPCR. Cq values of the mitochondrial gene *NADHdS1* were normalized to the nuclear gene *LPL* and expressed as relative mitochondrial DNA levels calculated by the 2^−ΔΔCt^ method. Results are expressed as mitochondrial DNA levels relative to brite negative control ± standard error of the mean (SEM) (n = 2). *p*-value: * *p* < 0.05. Abbreviations: NC (negative control), mitoDNA (mitochondrial DNA).

Mitochondrial DNA quantity decreased in white adipocyte control as compared to brite adipocyte control (−40.28% ± 4.17; *p* < 0.05), suggesting increased mitochondrial biogenesis in brite adipocytes and confirming effective adipocyte browning stimulation (Figure 6). Nevertheless, no statistically significant variation of mitochondrial DNA content was detected in brite adipocytes transfected with plant miR6262 mimic in comparison to brite control cells (Figure 6).

## 4. Discussion

Extensive evidence has reported the beneficial effects of plants on the restoration of energy balance and the counteraction of metabolic disturbances, including the stimulation of adipose tissue browning and the alleviation of steatosis and NAFLD [2,4,6]. Based on this evidence and the emergence of plant miRNAs as cross-kingdom regulators with bioactive effects in animals [17], the present article ought (1) to identify plant miRNAs that may influence the expression of crucial genes of hepatocytes and adipocytes involved in metabolism and thermogenesis, (2) to evaluate their relevance on lipid accumulation and the browning process.

The in silico approach revealed that miR6262, which was first identified in peach [67], could potentially regulate the expression of the human *RXR* gene. This gene encodes for a protein that is part of the retinoid X receptor family, which is located at the nucleus and has a key role in the regulation of cell proliferation, death and differentiation, and glucose, fatty acid and cholesterol metabolism [65,68,69]. We hypothesized that plant miR6262 might improve the metabolic gene expression profile of hepatocytes and adipocytes and could promote the expression of adipocyte thermogenic markers, through the eventual interaction with *RXRA*, which could presumably be directed at inhibiting their expression [20]. The basis of this hypothesis relies on the evidence supporting (1) the therapeutic potential of plants to promote energy expenditure and counteract metabolic disturbances through the alleviation of liver and WAT dysfunction and the activation of BAT [4,6,8]; (2) the key role of RXR receptors in liver and adipocyte physiology [65,69]; (3) the association between the activation of RXR or signaling pathways in which RXR function is involved and the inhibition of adipocyte browning, development of dysfunctional WAT, and exacerbation of hepatic steatosis [64,70]. In addition, reported evidence has shown an association between RXR inhibition and the protection against obesity, and associated comorbidities such as diabetes, by improving metabolic homeostasis and enhancing energy expenditure [65,66].

This work provides the first insight into the impact of plant miR6262 on the downregulation of the expression of the predicted human target *RXRA* in both human adipocytes and hepatocytes. Notably, the impact of plant miR6262 on gene expression metabolic profile on both cell types was different.

On the one hand, in human hepatocytes, plant miR6262 influenced the mRNA levels of transcription factors and protein-coding genes linked to lipid and glucose metabolism. miR6262 promoted an extensive inhibition of *RXRA* and *PPARA* mRNA levels in basal (untreated with FFA) cells and HepG2 cells exposed to FFA to mimic hepatic steatosis. RXRα heterodimerizes with PPARα [70,71], which might explain why the downregulation of *RXRA* expression by miR6262 could affect negatively *PPARA* mRNA levels. *RXRA* and *PPARA* inhibition was accompanied by a downregulation in *SREBF1* mRNA expression, which could correlate with the evidence confirming that RXRα and PPARα heterodimers stimulate the expression of the key inductor of lipogenesis *SREBF1* [70,72,73]. However, miR6262 did not downregulate *FASN* mRNA expression in HepG2 cells exposed to FFA, an effect that was observed in non-FFA-treated HepG2. This observation might be associated with the higher inhibition of *SREBF1* in the baseline situation as compared to the FFA-overloading state. A remarkable effect promoted by miR6262 was a great decrease of *G6PC* mRNA levels, which encodes for a glucose 6-phosphatase, a crucial enzyme involved in the production of liver endogenous glucose [74,75]. *G6PC* (also termed *G6Pase*) is directly regulated by PPARα and, in agreement with our results, downregulation of *PPARA* and/or *RXRA* could be accompanied by a decrease in *G6PC* expression [66,76]. Nevertheless, the exact mechanism underlying interaction and temporal dynamics between the potential genes modulated by miR6262 should be confirmed and further evaluated in future studies. Overall, these results suggest that miR6262 could be a bioactive molecule from plant origin that, as well as plant miR8126, could influence the expression profile of crucial human hepatic genes associated with glucose and lipid metabolism [39]. However, gene expression changes in the metabolic profile promoted by plant miR6262 did not translate into the attenuation of triglyceride accumulation in the in vitro model of liver steatosis. Despite using two independent techniques (Nile Red staining and Triglyceride-Glo^TM^ Assay), no variation in the lipid content was detected in miR6262-transfected hepatocytes that were treated with FFA.

On the other hand, in brown-like adipocytes, plant miR6262 decreased *PLIN1* gene expression, which encodes for perilipin-1, a key regulator of lipolysis [77,78]. Also, miR6262 upregulated *CIDEA* and *CPT1M* (also termed *CPT1B*), which are key markers of the browning process, involved in thermogenesis and energy expenditure [51,52,79]. In agreement with our results, other studies have found an association between RXR inhibition and the increase of *CIDEA* expression [64]. Nevertheless, the upregulation of *CIDEA* expression by miR6262 did not correlate with an increase of *UCP1* mRNA levels, despite the extensive evidence showing that *CIDEA* stimulates *UCP1* expression, the main driver of the browning process [79,80,81]. These results correlated with the evaluation of UCP-1 protein levels, which revealed that miR6262 did not affect UCP-1 expression; even a trend towards the downregulation was observed. In addition, miR6262 did not exert a functional impact on mitochondriogenesis, which is linked to the browning process [56,57]. These might suggest that plant miR6262 might specifically affect key players in adipocytes browning process although it seems unlikely that this might translate to a functionally relevant biological impact in this cell type.

The cross-kingdom xenomiRs hypothesis is still a matter of debate. In agreement with our results concerning plant miR6262 functional effects, other authors have reported that certain dietary xenomiRs, despite being efficiently uptaken by mammalian cells, might not be functional in mammals [82,83]. By contrast, some studies have reported that plant miRNAs could exert a biological impact on adipocytes, influencing adipogenesis [37], and improving the inflammatory and metabolic profile in obese mice [36]. Although we could not determine a cross-kingdom biological activity for plant miR6262 on counteracting lipid accumulation in hepatocytes and stimulating the browning process in adipocytes, in the conditions of our study, we cannot confirm that plant miR6262 could not be functional in these cell types. In this context, it might entail special relevance to analyze the effect of miR6262 on hepatocyte gluconeogenesis, based on (1) the remarkable downregulation of *G6PC* mRNA promoted by miR6262 reported in the present study, and (2) the fact that liver gluconeogenesis could be suppressed by targeting *G6PC* which improves liver and adipose tissue metabolic profile, insulin sensitivity and diet-induced obesity [74,75]. It would be also important to evaluate the functional impact of miR6262 on adipocyte lipolysis, given that it modulated *PLIN1* gene expression and Perilipin proteins prevent lipase activity thus acting as master regulators of this metabolic process [84]. In addition, while the effect of plant miRNAs should first be evaluated individually to validate miRNA-target gene predictions and elucidate mechanisms of action, it could be important to consider that plants may contain a wide range of miRNAs with potential cross-kingdom regulatory activities [18]. Therefore, evaluating the physiological impact of a cocktail of plant miRNAs with similar potential phenotypic effects, such as miR6262 and miR8126 isoforms, could be relevant and represent a more physiological setting as compared to human plant miRNAs ingestion from food [39]. This fact is important because additive and/or synergistic effects between plant miRNAs may occur, potentially leading to metabolic phenotypic changes that might not be observed, or not to the same extent, when miRNAs are administered individually [85].

Certain limitations should be acknowledged in this study: (1) Several reports suggest that many plant miRNAs are evolutionarily conserved across species [86,87]. In this study, the *Prunus persica* genome was selected as the reference for identifying plant miRNAs with potential human targets related to metabolism, leading to the selection of miR6262 as a candidate. However, it would be especially relevant to analyze miR6262 expression across a broader range of plant-based products to determine whether this plant microRNA might be specific to peaches or conserved throughout the plant kingdom (and to what extent). (2) Further research is needed to determine whether the miR6262 effects on metabolic and thermogenic gene expression profile might eventually translate into phenotypic changes beyond hepatocyte lipid accumulation or adipocyte browning. In this context, RNA-sequencing analysis could contribute to underpinning the miR6262 mechanism of action and target gene interactions. (3) The bioavailability of plant miRNAs in animal circulatory systems still remains controversial due to inconsistent results [41]. Thus, it will be necessary to evaluate whether plant miR6262 could be absorbed to eventually reach specific tissues and organs, and eventually modulate cell function. (4) In this regard, in vivo experiments will be crucial to elucidate the physiological impact and therapeutic potential of miR6262. However, genomic differences between species may arise, so careful consideration would be required when selecting an appropriate animal model. Despite these caveats, the results of this study pave the way for further investigation into the cross-kingdom biological impact of plant miR6262, suggesting a promising role of this microRNA in the regulation of metabolism and thermogenesis.

## 5. Conclusions

The present study reveals that plant miR6262 could be a bioactive molecule capable of modulating the expression of human genes associated with energy expenditure and glucose and lipid metabolism in hepatocytes and adipocytes. Nevertheless, we did not find evidence that plant miR6262 could exert a biological function in hepatocytes and adipocytes related to the counteraction of lipid accumulation or adipocyte browning stimulation, at least in our experimental conditions. Thus, the functional impact of plant miR6262 remains elusive, and further experiments will be necessary to determine whether the gene expression changes promoted by this miRNA could entail biological relevance in humans. Notwithstanding, these findings provide a new avenue for further research into the role of plant miR6262 as a cross-kingdom gene expression regulator in the context of metabolism.

## Figures and Tables

**Table 1:** Predesigned qPCR from Taqman (Thermo Fisher Scientific Inc.) (^1^) and Integrated DNA Technologies (^2^) primers used for gene expression analyses.

Gene Name	Assay ID	Accession Number (RefSeq)
*ACOX1*	^2^ Hs.PT.56a.3058584	NM_001185039(3)
*FASN*	^1^ Hs01005622_m1	NM_004104.4; XM_011523538.2
*FOXO1*	^2^ Hs.PT.58.40005627	NM_002015(1)
*G6PC*	^2^ Hs.PT.58.5006581	NM_000151(2)
*GSK3B*	^2^ Hs.PT.58.40111551	NM_001146156(2)
*MAPKAPK2*	^2^ Hs.PT.58.2443418	NM_004759(2)
*PPARA*	^2^ Hs.PT.58.45310483	NM_001001928(2)
*QKI*	^2^ Hs.PT.58.2815647	NM_006775(1)
*RXRA*	^2^ Hs.PT.58.3784663	NM_002957(1)
*SREBF1*	^2^ Hs.PT.58.3359761	NM_001005291(2)
*TBP*	^1^ Hs00427620_m1	NM_001172085.1; NM_003194.4

Gene names, assays IDs and accession number of predesigned oligonucleotide sequences from Taqman (Thermo Fisher Scientific Inc., Waltham, MA, USA) and Integrated DNA Technologies (IDT; Coralville, IA, USA). Abbreviations: ID (identification), *ACOX1* (Acyl-CoA Oxidase 1), *FASN* (Fatty Acid Synthase), *FOXO1* (Forkhead Box O1), *G6PC* (Glucose-6-Phosphatase Catalytic Subunit 1), *GSK3B* (Glycogen Synthase Kinase 3 Beta), *MAPKAPK2* (MAPK Activated Protein Kinase 2), *PPARA* (Peroxisome Proliferator Activated Receptor Alpha), *QKI* (Quaking Homolog, KH Domain RNA Binding), RefSeq (Reference Sequence), *RXRA* (Retinoid X Receptor Alpha), *SREBF1* (Sterol Regulatory Element Binding Transcription Factor 1), *TBP* (TATA-box binding protein).

**Table 2:** Primers designed for qPCR mRNA gene expression analysis.

Gene Name	Forward Primer	Reverse Primer
*ACOX1*	TCTTCACTTGGGCATGTTCCT	TTCCAGGCGGGCATGA
*ADRB3*	GCCTTCGCCTCCAACATG	GCATCACGAGAAGAGGAAGG
*ATGL* (*PNPLA2*)	GGGAGAAGATCACGTCCTGG	CTCCAGCAAGCAGATGGTGA
*CIDEA*	GCGAGAGTCACCTTCGACTTG	CGTTAAGGCAGCCGATGAA
*COL1A1*	ACCTGCGTGTACCCCACTCA	CCGCCATACTCGAACTGGAA
*CPT1M* (*CHKB-CPT1B*)	AACTCCATAGCCATCATCTGCT	GAGCAGCACCCCAATCAC
*FABP4*	TGTGCAGAAATGGGATGGAAA	CAACGTCCCTTGGCTTATGCT
*HSL* (*LIPE*)	GCACTACAAACGCAACGAGACA	GGTTCTGTGTGATCCGCTCAA
*PLIN1*	ACCCCCCTGAAAAGATTGCTT	GATGGGAACGCTGATGCTGTT
*PPARG 1+2*	AGCCTCATGAAGAGCCTTCCA	TCCGGAAGAAACCCTTGCA
*PPARG2*	CAAACCCCTATTCCATGCTGTT	ATCAGTGAAGGAATCGCTTTCTG
*36B4* (*RPLP0*)	AGGCAGATGGATCAGCCAAGA	TGCATCAGTACCCCATTCTATCAT
*UCP1*	GTGTGCCCAACTGTGCAATG	CCAGGATCCAAGTCGCAAGA

Gene names, forward and reverse oligonucleotide sequences designed with Primer Express Software (Perkin Elmer and Analytical Sciences, Boston, MA, USA; http://www.perkinelmer.com). Abbreviations: *ACOX1* (Acyl-CoA Oxidase 1), *ADRB3* (Adrenoceptor Beta 3), *ATGL* (Adipose Triglyceride Lipase), *CIDEA* (Cell Death Inducing DFFA Like Effector A), *COL1A1* (Collagen Type I Alpha 1 Chain), *CPT1M* (Carnitine palmitoyltransferase I), *FABP4* (Fatty Acid Binding Protein 4), *HSL* (Hormone-sensitive lipase), *PLIN1* (Perilipin 1), *PPARG* (Peroxisome Proliferator-Activated Receptor Gamma), *RPLP0* (Ribosomal Protein Lateral Stalk Subunit P0), *UCP1* (Uncoupling Protein 1).

**Table 3:** Primers designed for qPCR mitochondrial DNA quantification.

Gene Name	Forward Primer	Reverse Primer
*LPL*	CGAGTCGTCTTTCTCCTGATGAT	TTCTGGATTCCAATGCTTCGA
*NADHdS1*	CCCTAAAACCCGCCACATCT	GAGCGATGGTGAGAGCTAAGGT

Gene names, forward and reverse oligonucleotide sequences generated using Primer Express Software (Perkin Elmer and Analytical Sciences, Boston, MA, USA; http://www.perkinelmer.com). Abbreviations: *LPL* (Lipoprotein Lipase), *NADHdS1* (NADH dehydrogenase Subunit 1).

**Table 4:** Predicted human target transcripts of plant miR6262 identified with the psRNATarget (scoring schemas V1 and V2) and TAPIR bioinformatic tools.

miR6262 Predicted Human Target Transcripts				
psRNATarget. Scoring Schema V1				
Target Accession	Expectation	UPE	mRNA Target Aligned Fragment (5′-3′)	Inhibitory Effect
**NM_020841|*OSBPL8***	1.5	14.479	3084-[GCAGUUUUAACUUUCUGAAGA]-3104	Cleavage
**NM_175854|*PAN3***	2.0	10.514	290-[CUAAUUUUAAUUUUUUAAAGA]-310	Cleavage
**NM_001695|*ATP6V1C1***	2.0	15.648	3909-[GUAAUUCUUGCUUUCUAAAGA]-3929	Cleavage
**NM_002957|*RXRA***	2.0	19.619	2544-[ACAAUCUUUAAUUUUCUAAAGA]-2565	Cleavage
**NM_003272|*GPR137B***	2.5	13.138	212-[AUAAUUUAAACUUUUUAAAGA]-232	Cleavage
**NM_001040424|*PRDM15***	2.5	14.964	2116-[ACAAUUUUAUUUUUUUAAAGA]-2136	Cleavage
**NM_033505|*EPT1***	2.5	11.079	2921-[UUAAUUCUAAUUUUCAAAAGA]-2941	Cleavage
**NM_205852|*CLEC12B***	2.5	14.003	1998-[ACAAGUAUAAUUUUCUAAAGA]-2018	Cleavage
**NM_001122842|*NCOA7***	3.0	15.696	763-[AUAAUUCUAAAUUUCUAAAAA]-783	Translation
**NM_004707|*ATG12***	3.0	16.889	2864-[CAGAUUUUAACUUUUUAAAGG]-2884	Cleavage
**NM_001277783|*ATG12***	3.0	16.889	2925-[CAGAUUUUAACUUUUUAAAGG]-2945	Cleavage
**NM_001172698|*PPARGC1B***	3.0	9.862	6938-[UAAAUUUUAAUUUUUUAAAGG]-6958	Cleavage
**NM_005282|*GPR4***	3.0	21.652	708-[GCAAUUCUAAGUUUCUAGAUA]-728	Translation
**NM_153261|*CNEP1R1***	3.0	14.936	1193-[ACAAUUCUCACUGUUUAGAGA]-1213	Translation
**NM_007203|*PALM2-AKAP2***	3.0	15.903	3119-[ACCCUUUUAACUUUCUGAAGA]-3139	Cleavage
**NM_021089|*ZNF8***	3.0	19.35	102-[UGACUUCUGACUUUCUAAGGA]-122	Cleavage
**psRNATarget. Scoring Schema V2**				
**Target accession**	**Expectation**	**UPE**	**mRNA target aligned fragment (5′-3′)**	**Inhibitory effect**
**NM_020841|*OSBPL8***	1.5	N/A	3084-[GCAGUUUUAACUUUCUGAAGA]-3104	Cleavage
**NM_175854|*PAN3***	1.5	N/A	290-[CUAAUUUUAAUUUUUUAAAGA]-310	Cleavage
**NM_003272|*GPR137B***	2.0	N/A	212-[AUAAUUUAAACUUUUUAAAGA]-232	Cleavage
**NM_001695|*ATP6V1C1***	2.0	N/A	3909-[GUAAUUCUUGCUUUCUAAAGA]-3929	Cleavage
**NM_033505|*EPT1***	2.0	N/A	2921-[UUAAUUCUAAUUUUCAAAAGA]-2941	Cleavage
**NM_004707|*ATG12***	2.0	N/A	2864-[CAGAUUUUAACUUUUUAAAGG]-2884	Cleavage
**NM_001277783|*ATG12***	2.0	N/A	2925-[CAGAUUUUAACUUUUUAAAGG]-2945	Cleavage
**NM_001172698|*PPARGC1B***	2.0	N/A	6938-[UAAAUUUUAAUUUUUUAAAGG]-6958	Cleavage
**NM_021089|*ZNF8***	2.0	N/A	102-[UGACUUCUGACUUUCUAAGGA]-122	Cleavage
**NM_205852|*CLEC12B***	2.5	N/A	1998-[ACAAGUAUAAUUUUCUAAAGA]-2018	Cleavage
**NM_001040424|*PRDM15***	3.0	N/A	2116-[ACAAUUUUAUUUUUUUAAAGA]-2136	Cleavage
**NM_002957|*RXRA***	3.0	N/A	2544-[ACAAUCUUUAAUUUUCUAAAGA]-2565	Cleavage
**NM_001122842|*NCOA7***	3.0	N/A	763-[AUAAUUCUAAAUUUCUAAAAA]-783	Translation
**NM_003034|*ST8SIA1***	3.0	N/A	5391-[AUGACUCUAACUUUUUAAAGC]-5411	Cleavage
**NM_024685|*BBS10***	3.0	N/A	1219-[GUAAUUCUGAUUUUUUAAACA]-1239	Cleavage
**NM_021183|*RAP2C***	3.0	N/A	1992-[AUGAUUUAAAUUUUCUAGAGA]-2012	Cleavage
**NM_007203|*PALM2-AKAP2***	3.0	N/A	3119-[ACCCUUUUAACUUUCUGAAGA]-3139	Cleavage
**NM_016072|*GOLT1B***	3.5	N/A	1813-[AUAAUUCUACCUUUUUAGAGC]-1833	Cleavage
**NM_001206866|*IL6R***	3.5	N/A	22-[ACAAUGCUAAUUUUUUAAAAA]-42	Cleavage
**NM_005282|*GPR4***	3.5	N/A	708-[GCAAUUCUAAGUUUCUAGAUA]-728	Translation
**NM_001256105|*WNT5A***	3.5	N/A	2300-[ACAAUCCUAGCUUUUAAAAGA]-2320	Cleavage
**NM_153261|*CNEP1R1***	4.0	N/A	1193-[ACAAUUCUCACUGUUUAGAGA]-1213	Cleavage
**NM_012089|*ABCB10***	4.0	N/A	188-[AUAAUUGUAACUUUUUAAAUG]-208	Cleavage
**NM_001042543|*GLRA3***	4.5	N/A	1598-[ACAAUUGUAAUUUUUUAAAAU]-1618	Cleavage
**TAPIR**				
**Target accession**	**Score**	**MFE ratio**	**mRNA target aligned fragment (5′-3′)**	
**NM_205852|*CLEC12B***	3.0	0.75	1998-[ACAAGUAUAAUUUUCUAAAGA]-2018	
**NM_001695|*ATP6V1C1***	3.0	0.77	3909-[GUAAUUCUUGCUUUCUAAAGA]-3929	
**NM_020841|*OSBPL8***	2.5	0.93	3084-[GCAGUUUUAACUUUCUGAAGA]-3104	
**NM_002957|*RXRA***	2.5	0.78	2544-[ACAAUCUUUAAUUUUCUAAAGA]-2565	
**NM_003272|*GPR137B***	3.0	0.73	212-[AUAAUUUAAACUUUUUAAAGA]-232	
**NM_007203|*PALM2-AKAP2***	3.5	0.79	3119-[ACCCUUUUAACUUUCUGAAGA]-3139	
**NM_203464|*AK4***	4.0	0.72	1546-[ACAUUACUUACUUUCUGAAGA]-1566	

The cDNA library termed as “Homo sapiens (human), transcript, Human genomic sequencing project” (available at psRNATarget server) was aligned with the plant miR6262 mature sequence (5′-UCUUUAGAAAGUUAGAAUUGU-3′). Bolded outputs indicate putative targets identified across all the prediction algorithms. Abbreviations: MFE (minimum free energy), N/A (not applicable), UPE (unpaired energy).

## Data Availability

The original contributions presented in the study are included in the article/Supplementary Materials; further inquiries can be directed to the corresponding author.

## Supplementary Materials

The following supporting information can be downloaded at: https://www.mdpi.com/article/10.3390/nu16183146/s1, Table S1: Gene ontology (GO) enrichment analyses of putative human target genes of plant miR6262; Figure S1: In silico prediction alignments between plant miR6262 and putative target transcripts; Figure S2: Expression levels of plant miR6262 in HepG2 cells after mimic transfection; Figure S3: Evaluation of the effect of plant miRNA mimic miR6262 on the viability of HepG2 cells; Figure S4: Gene expression analyses of HepG2 cells transfected with plant miR6262 mimic.
