# Supplementary material for: Plant miR6262 Modulates the Expression of Metabolic and Thermogenic Genes in Human Hepatocytes and Adipocytes
Source: Nutrients. 2024 Sep 18;16(18):3146. doi: 10.3390/nu16183146 (PMC11435339; doi:10.3390/nu16183146)
Supplement: Supplementary file 1 [file nutrients-16-03146-s001.zip › nutrients-3192335-supplementary.pdf]

nutrients

MDPI

Article

Plant miR6262 modulates the expression of metabolic and thermogenic genes in human hepatocytes and adipocytes

Ester Díez-Sainz [1], Fermín I Milagro [1,2,3,*], Paula Aranaz [1,2], José I Riezu-Boj [1,2] and Silvia Lorente-Cebrián [4,5,6]

[1] Department of Nutrition, Food Science and Physiology/Center for Nutrition Research, Faculty of Pharmacy and Nutrition, University of Navarra, 31008 Pamplona, Spain; ediezsainz@alumni.unav.es (E.D.-S.); paranaz@unav.es (P.A.); jiriezu@unav.es (J.I.R.-B.); fmilagro@unav.es (F.I.M.)
[2] Navarra Institute for Health Research (IdiSNA), 31008 Pamplona, Spain
[3] Centro de Investigación Biomédica en Red Fisiopatología de la Obesidad y Nutrición (CIBERobn), Instituto de Salud Carlos III, 28029 Madrid, Spain
[4] Department of Pharmacology, Physiology and Legal and Forensic Medicine, Faculty of Health and Sport Science, University of Zaragoza, 50009 Zaragoza, Spain.; slorentec@unizar.es (S.L.-C.)
[5] Instituto Agroalimentario de Aragón-IA2, Universidad de Zaragoza-CITA, 50013 Zaragoza, Spain
[6] Aragón Health Research Institute (IIS-Aragon), 50009 Zaragoza, Spain
* Correspondence: fmilagro@unav.es

Supplementary Materials

Table S1. Gene ontology (GO) enrichment analyses of putative human target genes of plant miR6262.

Plant miR6262 putative target genes			
**Biological Process**	**Relative enrichment**	**Adjusted p value**	**Genes**
Mitochondrial Unfolded Protein Response (Go:0034514)	375.06	0.035	*ABCB10*
Positive Regulation Of Heme Biosynthetic Process (Go:0070455)	250.04	0.035	
Positive Regulation Of Haemoglobin Biosynthetic Process (Go:0046985)	125.02	0.038	
Regulation Of ATP Biosynthetic Process (Go:2001169)	375.06	0.035	*AK4*
ADP Biosynthetic Process (Go:0006172)	150.03	0.036	
Amp Metabolic Process (Go:0046033)	107.16	0.038	
Nucleobase-Containing Small Molecule Interconversion (Go:0015949)	93.77	0.040	
Nucleoside Triphosphate Biosynthetic Process (Go:0009142)	75.01	0.044	
Nucleoside Monophosphate Phosphorylation (Go:0046940)	68.19	0.045	
Regulation Of Oxidative Phosphorylation (Go:0002082)	57.70	0.048	
GTP Metabolic Process (Go:0046039)	53.58	0.048	
Positive Regulation Of Viral Translation (Go:1904973)	250.04	0.035	*ATG12*
C-Terminal Protein Lipidation (Go:0006501)	150.03	0.036	
Regulation Of Autophagosome Maturation (Go:1901096)	125.02	0.038	
Autophagy Of Nucleus (Go:0044804)	107.16	0.038	
Negative Regulation Of Defence Response To Virus (Go:0050687)	53.58	0.048	
Natural Killer Cell Inhibitory Signalling Pathway (Go:0002769)	250.04	0.035	*CLEC12B*
Negative Regulation Of Receptor Signalling Pathway Via Stat (Go:1904893)	83.35	0.043	
Negative Regulation Of Natural Killer Cell Mediated Cytotoxicity (Go:0045953)	57.70	0.048	
Melanocyte Proliferation (Go:0097325)	300.05	0.006	*CLEC12B, WNT5A*

Positive Regulation Of Triglyceride Biosynthetic Process (Go:0010867)	53.58	0.048	*CNEP1R1*
Phosphatidylethanolamine Biosynthetic Process (Go:0006646)	75.01	0.044	*EPT1*
Synaptic Transmission, Glycinergic (Go:0060012)	150.03	0.036	*GLRA3*
Positive Regulation Of Protein Localization To Lysosome (Go:0150032)	375.06	0.035	*GPR137B*
Regulation Of Macrophage Activation (Go:0043030)	68.19	0.045	
Glomerular Mesangial Cell Development (Go:0072144)	187.53	0.036	*GPR4*
Angiogenesis Involved In Wound Healing (Go:0060055)	75.01	0.044	
Regulation Of Vascular Permeability (Go:0043114)	57.70	0.048	
Response To Acidic Ph (Go:0010447)	53.58	0.048	
Positive Regulation Of Inflammatory Response (Go:0050729)	15.00	0.038	*GPR4, WNT5A*
Hepatic Immune Response (Go:0002384)	375.06	0.035	*IL6R*
Interleukin-11-Mediated Signalling Pathway (Go:0038154)	250.04	0.035	
Positive Regulation Of Glomerular Mesangial Cell Proliferation (Go:0072126)	150.03	0.036	
Ciliary Neurotrophic Factor-Mediated Signalling Pathway (Go:0070120)	150.03	0.036	
Vascular Endothelial Growth Factor Production (Go:0010573)	107.16	0.038	
T-Helper 17 Cell Lineage Commitment (Go:0072540)	107.16	0.038	
Positive Regulation Of Activation Of Janus Kinase Activity (Go:0010536)	107.16	0.038	
Positive Regulation Of Platelet Aggregation (Go:1901731)	68.19	0.045	
Neutrophil Mediated Immunity (Go:0002446)	68.19	0.045	
Negative Regulation Of Collagen Biosynthetic Process (Go:0032966)	57.70	0.048	
Positive Regulation Of Leukocyte Chemotaxis (Go:0002690)	53.58	0.048	
Interleukin-6-Mediated Signalling Pathway (Go:0070102)	50.01	0.050	
Positive Regulation Of Chemokine Production (Go:0032722)	31.26	0.035	*IL6R, WNT5A*
Positive Regulation Of Interleukin-6 Production (Go:0032755)	15.15	0.038	
Positive Regulation Of Nf-Kappab Transcription Factor Activity (Go:0051092)	9.56	0.048	
Negative Regulation Of Cellular Response To Oxidative Stress (Go:1900408)	250.04	0.035	*NCOA7*
Negative Regulation Of Peptidyl-Cysteine S-Nitrosylation (Go:1902083)	107.16	0.038	
Negative Regulation Of Oxidative Stress-Induced Neuron Death (Go:1903204)	53.58	0.048	
Protein Localization To Nuclear Pore (Go:0090204)	250.04	0.035	*OSBPL8*
Negative Regulation Of Sequestering Of Triglyceride (Go:0010891)	125.02	0.038	
Phosphatidylserine Acyl-Chain Remodelling (Go:0036150)	62.51	0.047	
Negative Regulation Of Cell Migration (Go:0030336)	9.62	0.048	*OSBPL8, RAP2C*
Activation Of Protein Kinase B Activity (Go:0032148)	48.40	0.035	*OSBPL8, WNT5A*
Positive Regulation Of Cytoplasmic mRNA Processing Body Assembly (Go:0010606)	125.02	0.038	*PAN3*
Nuclear-Transcribed mRNA Catabolic Process, Deadenylation-Dependent Decay (Go:0000288)	62.51	0.047	
Nuclear-Transcribed mRNA Poly(A) Tail Shortening (Go:0000289)	50.01	0.050	
Positive Regulation Of Alkaline Phosphatase Activity (Go:0010694)	125.02	0.038	*PPARGC1B*
Bone Trabecula Formation (Go:0060346)	93.77	0.040	
Mitochondrial Transcription (Go:0006390)	75.01	0.044	

Positive Regulation Of Transcription, DNA-Templated (Go:0045893)	4.09	0.047	*PPARGC1B, RXRA, RAP2C, WNT5A*
Regulation Of Stem Cell Division (Go:2000035)	75.01	0.044	*PRDM15*
Positive Regulation Of Transcription By RNA Polymerase II (Go:0045944)	3.20	0.048	*PRDM15, PPARGC1B, RXRA, NCOA7, WNT5A*
Regulation Of Protein Tyrosine Kinase Activity (Go:0061097)	250.04	0.035	*RAP2C*
Rap Protein Signal Transduction (Go:0032486)	68.19	0.045	
Establishment Of Endothelial Intestinal Barrier (Go:0090557)	53.58	0.048	
Positive Regulation Of Thyroid Hormone Mediated Signalling Pathway (Go:0002157)	250.04	0.035	*RXRA*
Positive Regulation Of Vitamin D Receptor Signalling Pathway (Go:0070564)	150.03	0.036	
Peroxisome Proliferator Activated Receptor Signalling Pathway (Go:0035357)	107.16	0.038	
Positive Regulation Of Transporter Activity (Go:0032411)	93.77	0.040	
Glycosphingolipid Biosynthetic Process (Go:0006688)	57.70	0.048	*ST8SIA1*
Planar Cell Polarity Pathway Involved In Outflow Tract Morphogenesis (Go:0061347)	750.13	0.035	*WNT5A*
Planar Cell Polarity Pathway Involved In Midbrain Dopaminergic Neuron Differentiation (Go:1904955)	750.13	0.035	
Planar Cell Polarity Pathway Involved In Cardiac Right Atrium Morphogenesis (Go:0061349)	750.13	0.035	
Tube Closure (Go:0060606)	750.13	0.035	
Planar Cell Polarity Pathway Involved In Pericardium Morphogenesis (Go:0061354)	750.13	0.035	
Planar Cell Polarity Pathway Involved In Cardiac Muscle Tissue Morphogenesis (Go:0061350)	750.13	0.035	
Planar Cell Polarity Pathway Involved In Ventricular Septum Morphogenesis (Go:0061348)	750.13	0.035	
Negative Regulation Of Cell Proliferation In Midbrain (Go:1904934)	750.13	0.035	
Chemoattraction Of Serotonergic Neuron Axon (Go:0036517)	750.13	0.035	
Axis Elongation (Go:0003401)	375.06	0.035	
Postsynapse Assembly (Go:0099068)	375.06	0.035	
Planar Cell Polarity Pathway Involved In Gastrula Mediolateral Intercalation (Go:0060775)	375.06	0.035	
Cervix Development (Go:0060067)	375.06	0.035	
Hypophysis Morphogenesis (Go:0048850)	375.06	0.035	
Chemorepulsion Of Dopaminergic Neuron Axon (Go:0036518)	375.06	0.035	
Lateral Sprouting Involved In Mammary Gland Duct Morphogenesis (Go:0060599)	375.06	0.035	
Positive Regulation Of Timing Of Anagen (Go:0051885)	375.06	0.035	
Convergent Extension (Go:0060026)	375.06	0.035	
Optic Cup Formation Involved In Camera-Type Eye Development (Go:0003408)	375.06	0.035	

Planar Cell Polarity Pathway Involved In Axon Guidance (Go:1904938)	250.04	0.035
Olfactory Bulb Interneuron Development (Go:0021891)	250.04	0.035
Regulation Of Branching Involved In Mammary Gland Duct Morphogenesis (Go:0060762)	250.04	0.035
Mesenchymal-Epithelial Cell Signalling (Go:0060638)	250.04	0.035
Planar Cell Polarity Pathway Involved In Axis Elongation (Go:0003402)	250.04	0.035
Mesodermal To Mesenchymal Transition Involved In Gastrulation (Go:0060809)	250.04	0.035
Primary Heart Field Specification (Go:0003138)	250.04	0.035
Convergent Extension Involved In Organogenesis (Go:0060029)	187.53	0.036
Convergent Extension Involved In Axis Elongation (Go:0060028)	187.53	0.036
Positive Regulation Of Heart Induction By Negative Regulation Of Canonical Wnt Signalling Pathway (Go:0090082)	187.53	0.036
Mammary Gland Branching Involved In Thelarche (Go:0060744)	187.53	0.036
Hindgut Morphogenesis (Go:0007442)	187.53	0.036
Negative Regulation Of Mesenchymal Cell Proliferation (Go:0072201)	187.53	0.036
Development Of Primary Male Sexual Characteristics (Go:0046546)	187.53	0.036
Epithelial Cell Proliferation Involved In Mammary Gland Duct Elongation (Go:0060750)	187.53	0.036
Negative Regulation Of Prostatic Bud Formation (Go:0060686)	187.53	0.036
Positive Regulation Of Protein Localization To Synapse (Go:1902474)	187.53	0.036
Urinary Bladder Development (Go:0060157)	187.53	0.036
Negative Regulation Of Synapse Assembly (Go:0051964)	150.03	0.036
Ameboidal-Type Cell Migration (Go:0001667)	150.03	0.036
Notochord Morphogenesis (Go:0048570)	150.03	0.036
Negative Regulation Of Melanin Biosynthetic Process (Go:0048022)	150.03	0.036
Wnt Signalling Pathway, Calcium Modulating Pathway (Go:0007223)	150.03	0.036
Positive Regulation Of Meiotic Nuclear Division (Go:0045836)	150.03	0.036
Non-Canonical Wnt Signalling Pathway Via Jnk Cascade (Go:0038031)	150.03	0.036
Positive Regulation Of Thymocyte Apoptotic Process (Go:0070245)	150.03	0.036
Wnt Signalling Pathway Involved In Midbrain Dopaminergic Neuron Differentiation (Go:1904953)	125.02	0.038
Paraxial Mesoderm Formation (Go:0048341)	125.02	0.038
Excitatory Synapse Assembly (Go:1904861)	107.16	0.038
Positive Regulation Of Response To Cytokine Stimulus (Go:0060760)	107.16	0.038
Positive Regulation Of Protein Kinase C Activity (Go:1900020)	107.16	0.038
Pericardium Morphogenesis (Go:0003344)	107.16	0.038
Anterior/Posterior Axis Specification, Embryo (Go:0008595)	93.77	0.040
Midgut Development (Go:0007494)	93.77	0.040
Cellular Response To Molecule Of Bacterial Origin (Go:0071219)	93.77	0.040
Positive Regulation Of Neuron Projection Arborization (Go:0150012)	93.77	0.040
Positive Regulation Of Non-Canonical Wnt Signalling Pathway (Go:2000052)	93.77	0.040
Midbrain Dopaminergic Neuron Differentiation (Go:1904948)	83.35	0.043
Atrial Septum Development (Go:0003283)	83.35	0.043

Primitive Streak Formation (Go:0090009)	83.35	0.043
Genitalia Development (Go:0048806)	75.01	0.044
Planar Cell Polarity Pathway Involved In Neural Tube Closure (Go:0090179)	75.01	0.044
Regulation Of Postsynaptic Cytosolic Calcium Ion Concentration (Go:0099566)	75.01	0.044
Vagina Development (Go:0060068)	75.01	0.044
Positive Regulation Of Protein Kinase C Signalling (Go:0090037)	75.01	0.044
Secondary Heart Field Specification (Go:0003139)	68.19	0.045
Positive Regulation Of Cell-Cell Adhesion Mediated By Cadherin (Go:2000049)	68.19	0.045
Inhibitory Synapse Assembly (Go:1904862)	62.51	0.047
Positive Regulation Of Macrophage Cytokine Production (Go:0060907)	57.70	0.048
Presynapse Assembly (Go:0099054)	53.58	0.048
Secondary Palate Development (Go:0062009)	53.58	0.048
Establishment Of Planar Polarity (Go:0001736)	50.01	0.050
Type B Pancreatic Cell Development (Go:0003323)	50.01	0.050
Digestive Tract Morphogenesis (Go:0048546)	50.01	0.050

Putative targets were identified with TAPIR and psRNATarget (scoring schema V1 and V1) and the annotation "GO Biological Process" of Genecodis4 was used to identify enriched pathways. The table shows biological processes of miR6262 putative target genes that reported an adjusted p value < 0.05.

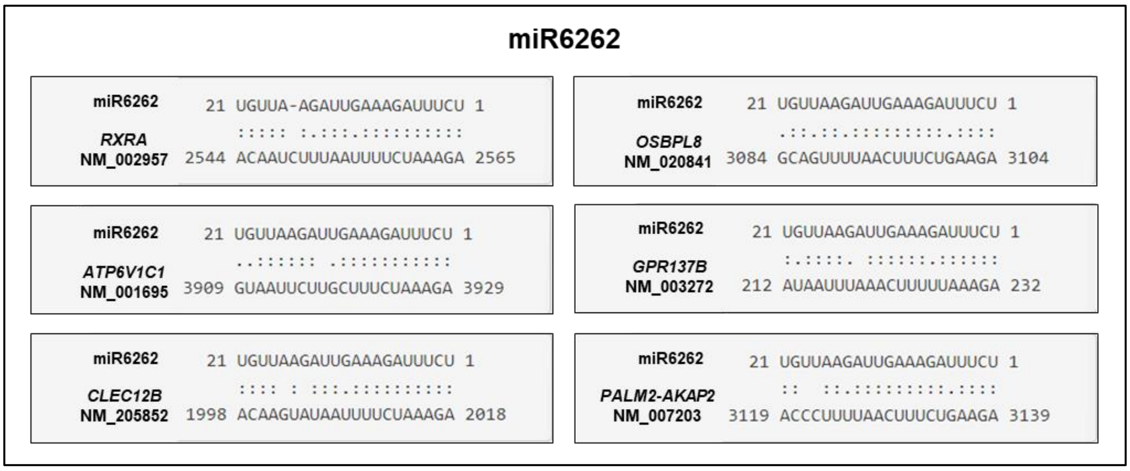

Figure S1. In silico prediction alignments between plant miR6262 and putative target transcripts. Alignments between plant miR6262 and the putative target transcripts of *RXRA, OSBPL8, ATP6V1C1, GPR137B, CLEC12B* and *PALM2-AKAP2* genes. The predictions algorithms psRNATarget (scoring schema V1 and V2) and TAPIR were used to identify putative human target transcript of the mature sequence of plant miR6262 (5’- UCUUUAGAAAGUUAGAAUUGU-3’) after the alignment with the cDNA library “Homo sapiens (human), transcript, Human genomic sequencing project” (from psRNATarget server). The Figure represents alignments between miR6262 and common outputs to the prediction algorithms used.

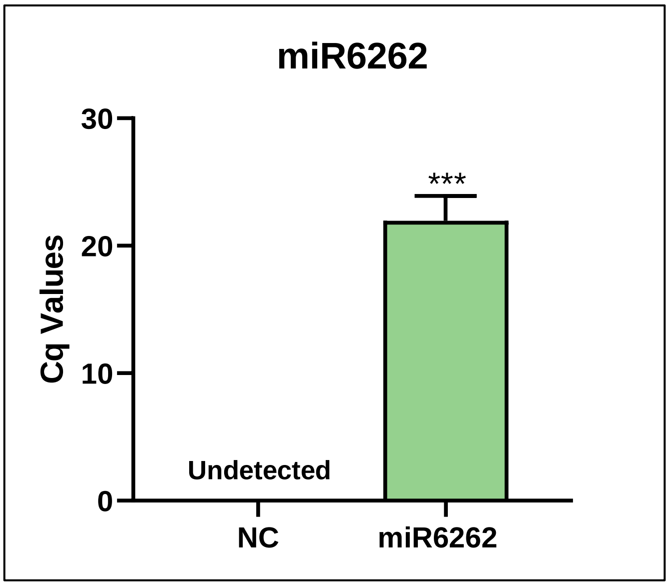

Figure S2. Expression levels of plant miR6262 in HepG2 cells after mimic transfection. HepG2 cells were transfected for 6 h with 50 nM of mirVana® mimic miR6262 (5′- UCUUUAGAAAGUUAGAAUUGU-3′) and a scramble sequence as a control (Negative Control). miRNA expression levels were evaluated by qPCR. Results are Cq values ± standard error of the mean (SEM) (n= 2-3). Significance refers to the comparison of each plant miRNA mimic respect to the control. p-value: *** p<0.001. Abbreviations: NC (negative control).

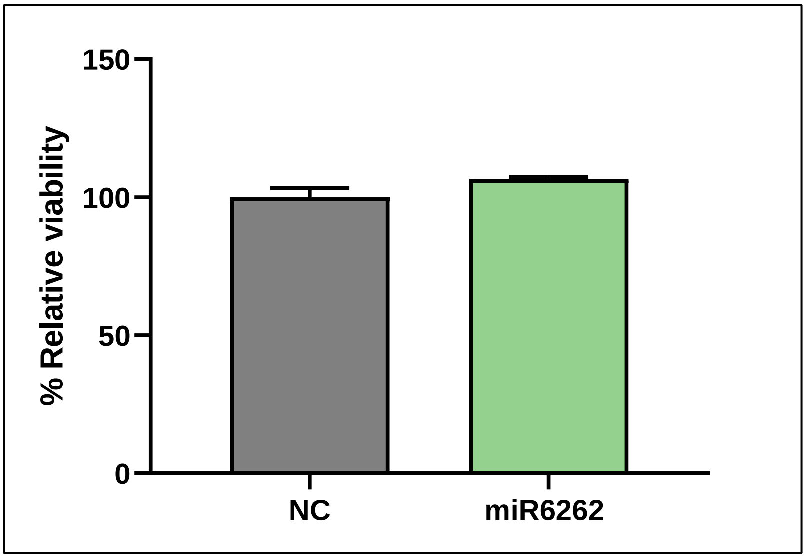

(a)

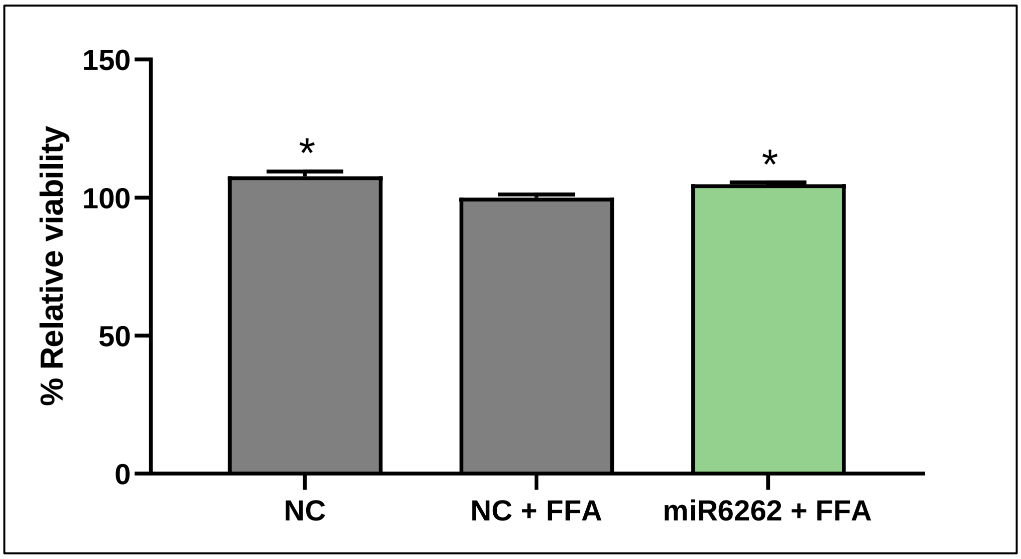

(b)

Figure S3. Evaluation of the effect of plant miRNA mimic miR6262 on the viability of HepG2 cells. (a) Relative viability of HepG2 cells transfected with miR6262. (b) Relative viability of HepG2 transfected with miR6262 and treated with free fatty acids. HepG2 cells were transfected for 48 h with 50 nM of mirVana mimic miR6262 (5′- UCUUUAGAAAGUUAGAAUUGU-3′) and a scramble sequence as a control (Negative Control). Additionally, 48 h after transfections cells were treated with 0.5 mM of free fatty acids (proportion oleic:palmitic acids 2:1) for 3 h until cell viability assessment. Cell viability was determined by MTS assay in control cells and cells transfected with plant miRNA mimics untreated and treated with fatty acids. For Figure S3a, results are presented as the % cell viability relative to the negative control ± standard error of the mean (SEM) (n= 4); and significance was assessed by establishing comparison between control cells and each plant miRNA mimic treatment. For Figure S3b, results are presented as the % cell viability relative to the negative control treated with fatty acids) ± standard error of the mean (SEM) (n= 3-4); and significance refers to the comparison of each plant miRNA mimic and untreated control cells respect to the control treated with fatty acids. p-value: * $p<0.05$. Abbreviations: NC (negative control), FFA (free fatty acids).

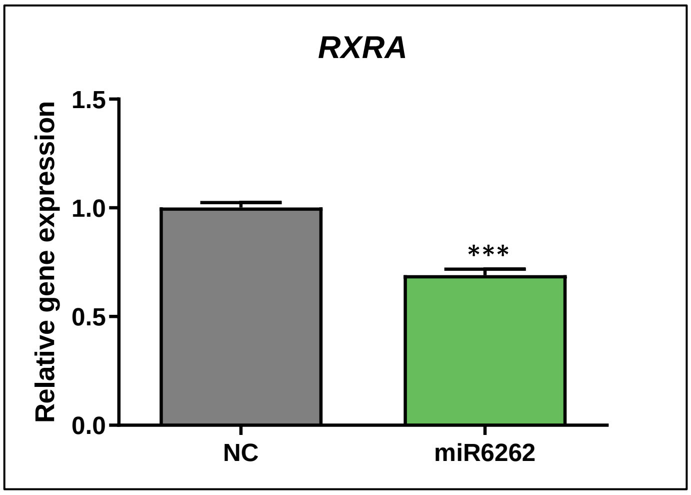

(a)

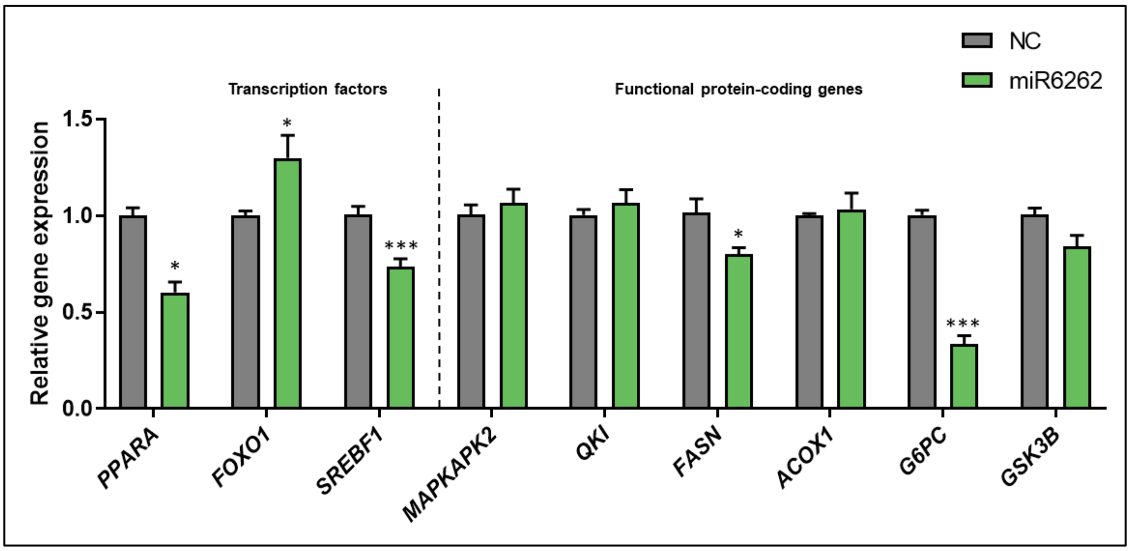

(b)

Figure S4. Gene expression analyses of HepG2 cells transfected with plant miR6262 mimic. (a) mRNA levels of the putative target gene *RXRA*. (b) mRNA levels of transcription factors (*PPARA*, *FOXO1*, and *SREBF1*) and functional protein coding genes (*MAPKAPK2, QKI, FASN, ACOX1, G6PC* and *GSK3B*) involved in glucose and lipid metabolism. HepG2 cells were transfected for 48 h with 50 nM of the mirVana mimic miR6262 (5′- UCUUUAGAAAGUUAGAAUUGU-3′) and a scramble sequence as a control (Negative Control). mRNA expression levels were quantified by qPCR. Cq values were normalized to the housekeeping gene *TBP* and expressed as the gene expression levels relative to the negative control cells, calculated by the $2^{-\Delta\Delta Ct}$ method. Results are presented as the relative gene expression mean ± standard error of the mean (SEM) (n= 2-7). p-value: * p<0.05, *** p<0.001. Abbreviations: NC (negative control).
